# Time Dependent Structure and Property Evolution in Fibres during Continuous Carbon Fibre Manufacturing

**DOI:** 10.3390/ma12071069

**Published:** 2019-04-01

**Authors:** Srinivas Nunna, Maxime Maghe, Rohit Rana, Russell J. Varley, Daniel B. Knorr, James M. Sands, Claudia Creighton, Luke C. Henderson, Minoo Naebe

**Affiliations:** 1 Carbon Nexus, Institute for Frontier Materials, Deakin University, Waurn Ponds, VIC 3216, Australia; snunna@deakin.edu.au (S.N.); maxime.mahge@deakin.edu.au (M.M.); rohit.rana@deakin.edu.au (R.R.); russell.varley@deakin.edu.au (R.J.V.); claudia.creighton@deakin.edu.au (C.C.); luke.henderson@deakin.edu.au (L.C.H.); 2 U.S. Army Research Lab, Composite and Hybrid Materials Branch, Aberdeen Proving Ground, Aberdeen, MD 21005, USA; daniel.b.knorr.civ@mail.mil (D.B.K.J.); james.m.sands.civ@mail.mil (J.M.S.)

**Keywords:** polyacrylonitrile fibres, carbon fibres, thermal stabilization, tensile properties, microstructure

## Abstract

Here we report on how residence time influences the evolution of the structure and properties through each stage of the carbon fibre manufacturing process. The chemical structural transformations and density variations in stabilized fibres were monitored by Fourier Transform Infrared Spectroscopy and density column studies. The microstructural evolution and property variation in subsequent carbon fibres were studied by X-ray diffraction and monofilament tensile testing methods, which indicated that the fibres thermally stabilized at longer residence times showed higher degrees of structural conversion and attained higher densities. Overall, the density of stabilized fibres was maintained in the optimal range of 1.33 to 1.37 g/cm^3^. Interestingly, carbon fibres manufactured from higher density stabilized fibres possessed lower apparent crystallite size (1.599 nm). Moreover, the tensile strength of carbon fibres obtained from stabilized fibres at the high end of the observed range (density: 1.37 g/cm^3^) was at least 20% higher than the carbon fibres manufactured from low density (1.33 g/cm^3^) stabilized fibres. Conversely, the tensile modulus of carbon fibres produced from low density stabilized fibres was at least 17 GPa higher than those from high density stabilized fibres. Finally, it was shown that there is potential to customize the required properties of resultant carbon fibres suiting specific applications via careful control of residence time during the stabilization stage.

## 1. Introduction

Carbon fibres are receiving increasing interest as the primary reinforcement in structural composites for automotive, aerospace, and renewable energy applications due to their ability to provide high specific strength and stiffness [1,2,3]. Carbon fibre can be manufactured from a variety of precursors such as polyacrylonitrile (PAN), pitch and rayon, although more than 90% of the current commercial carbon fibre market share is made using PAN based fibres [4,5,6]. During conversion of PAN to carbon fibres, PAN is initially thermally stabilized in an air atmosphere at temperatures between 200 and 300 °C. The fibre is then carbonized in an inert atmosphere typically up to 1600 °C in combination with various tensions and dwell times, depending on target properties and applications [2]. Despite extensive efforts over the past 50 years of research and development, the maximum tensile strength and modulus of commercial PAN based carbon fibres are still less than 10% and 50% of their theoretical values, respectively [7,8]. Though substantial work has been done to improve these properties through the use of additives such as CNTs [9], Graphene [10], and combinations of these with modified processing conditions [11,12] with good success.

The complexity of manufacturing, and the resulting high cost of this material currently hinders its extensive use in mainstream applications such as automobiles [13,14]. Thus, there is an enormous economic and academic driving force to both achieve desirable mechanical performance of carbon fibre (Tensile strength ~3.5 GPa, Tensile Modulus ~230 GPa) and reduce its cost. 

It is well known that temperature, time and tension are major parameters affecting the final properties during the manufacturing process [15,16]. A part from the precursor chemistry and manufacturing conditions, it has been reported that the stabilization of PAN fibres under optimum processing conditions not only assists in the efficient evolution of intermediate polymer structures within the fibre but also improves the final carbon fibre properties [17]. For instance, with the stabilization of PAN fibres at temperatures higher than their fusion temperature (275 °C) there is a significant effect on the tensile properties of carbon fibres because of the degradation of ladder polymer structures [17]. Moreover, excessive thermal stabilization leads to unwarranted incorporation of oxygen in the intermediate structures deleteriously affecting the properties of the resultant carbon fibres via the formation of pores [17,18]. Wu et al. [19] demonstrated that applying tension to the PAN fibres during the oxidation process has different effects at different temperatures. For instance, with an increase in tension from 1 N to 3 N on the fibres at lower temperatures ranging from (160 to 190 °C) during thermal stabilization showed a significant improvement in the tensile properties of final carbon fibres when compared to carbon fibres processed from stabilized fibres stretched at temperatures between (210 to 230 °C). There have been several studies [20,21,22,23] investigating the influence of process parameters on the structure and property evolution of stabilized and carbon fibres using a continuous step-wise process, which is a direct representation of the industrial production process. However, to the best of our knowledge, the majority of research is dedicated towards understanding the effect of varying combination of multiple process parameters on the structure-property development in stabilized and carbonized fibres. Thus, there is a need for a systematic and controlled study examining a single process condition, and how this manifests as structure/properties of the final carbon fibre.

There are some reports [19,24,25,26] that focus on investigating the influence of individual process parameters on fibre structure and morphology evolution using lab-scale furnaces. These studies have fundamental disconnects from the continuous, industrial scale manufacture of carbon fibres that make translation of those studies to meaningful advancements in carbon fibre manufacture challenging. Moreover, in order to improve the properties of carbon fibres, one needs to understand the effect of each individual parameter involved in the process.

Hence, in the current study, we have used a pilot scale carbon fibre manufacturing line to present a systematic approach to understand the effect of dwell time on the structure and property transformations of stabilized PAN fibres and also the resulting carbon fibres after low and high temperature carbonisation. By altering the line speed, various fibre samples were prepared and collected from each zone corresponding to each stage of the manufacturing process. Fibres from stabilization were assessed to understand the chemical structural transformations and density variations. Furthermore, the carbonized fibres were examined to understand the micro-structure and property variations in correlation with dwell times. Finally, an understanding of how the stabilized fibres dictate the structure of the final carbon fibres was developed for various residence times.

## 2. Materials and Methods 

A commercial PAN precursor with a tow size of 12,000 filaments (12 K) was used for this study. The physical properties of the precursor fibres are shown in Table 1.

### 2.1. Sample Preparation

**Figure 1:**
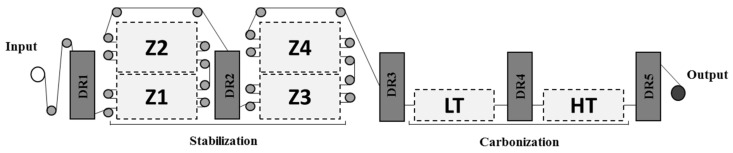
Schematic representation of the carbon fibre production line at Carbon Nexus, Deakin University.

Various industrial trials were conducted using the state-of-the-art carbon fibre production line at Carbon Nexus, Deakin University (Victoria, Australia). The manufacturing process is schematically presented in Figure 1. Four sets of samples were obtained at different dwell times by varying the overall production line speed. The temperature and tension at each sub-stage of the process were held constant throughout the study. The corresponding process parameters used for this study are listed in Table 2 with the sample codes given being based on the total dwell time spent in stabilization ovens. For example, sample code CF-72 is assigned to the sample that spent 72 min in the stabilisation ovens (18 min in each of the 4 ovens) at a 20 m/h line speed. Moreover, the process conditions chosen for this study were based on our previous experiences with the manufacture of PAN precursors.

### 2.2. Fourier Transform Infrared Spectroscopy (FTIR)

FTIR spectroscopic analysis was conducted on fibre samples using a Bruker Lumos FTIR (Bremen, Germany) equipped with a germanium crystal in the attenuated total reflectance (ATR) mode. For each sample, at least five measurements along the fibre bundle were acquired between 600 and 4000 cm^−1^ using an average of 128 scans at a resolution of 4 cm^−1^. Before each sample a background data was collected using the same acquisition parameters. The extent of the stabilization was quantified by calculating the cyclization and the dehydrogenation indices using the following equations described elsewhere [16,27]:

(1) $$\mathrm{Cyclization}\;\mathrm{Index}\;\left(\%\right)=\;\frac{\left(100\times \;0.29\;\times \;\mathrm{Abs}\;\left(1590\right)\right)}{((\mathrm{Abs}\left(2242\right)+\left(\;0.29\;\times \;\mathrm{Abs}\;\left(1590\right)\right)}$$

(2) $$\mathrm{Dehydrogenation}\;\mathrm{index}=\frac{\mathrm{Abs}\;\left(1360\right)}{\mathrm{Abs}\;\left(1450\right)}$$

where Abs (1590) and Abs (2242) are the measured absorbance (Abs) of the C=N and C≡N groups, Abs (1360) and Abs (1450) represents the intensity corresponds to CH and CH_2_ functional groups, respectively.

### 2.3. X-ray Diffraction Studies (XRD)

X-ray diffraction studies were conducted on carbon fibre samples using PANALYTICAL XPert Powder XRD instrument (Almelo, The Netherlands) equipped with a CuK α radiation source. The wavelength of the source was 1.540598 Å. An operating voltage 40 V and current 30 mA were considered for data collection. The fibres were initially cut and placed on a low noise silicon substrate and scanned between 10° and 60° diffraction angles. Scanning was conducted at a step size of 0.0130 and a time step of 150 s was used. The crystallite size of each fibre sample was calculated using Scherrer’s equation.

### 2.4. Density Measurements

The mass density of PAN precursor fibres, stabilized PAN fibres and subsequent carbon fibres were measured at 23 °C using a density gradient column method in accordance with ASTM D1505-10. Two density columns were used, where the first column contains a mixture of potassium iodide and distilled water solution with a gradient range of 1.17 to 1.45 g/cm^3^ and used for acquiring densities of precursor and stabilized fibres. The other column consists of a solution mixture of 3-ethylphosphate and 1,3-dibromopropane having a density gradient of 1.60 to 1.90 g/cm^3^ and used for obtaining density of carbon fibres.

### 2.5. Tensile Properties

The mechanical properties of fibre samples were obtained using a Textechno Favimat monofilament testing machine (Favitmat, Textechno, Mönchengladbach, Germany). The instrument was equipped with a 210 cN load cell and clamps which had a surface area of 4 mm × 4 mm. Tensile properties of stabilized PAN fibres were obtained at a test speed of 8 mm/min whereas a test speed of 2 mm/min was used for carbon fibre samples. A pretension of 0.5 cN and a gauge length of 25 mm were used for all the samples. At least 50 filaments were tested at each stage of the carbon fibre manufacturing process.

## 3. Results

### 3.1. Influence of Dwell Time on the Progress of Thermal Stabilization in PAN Fibres

**Figure 2:**
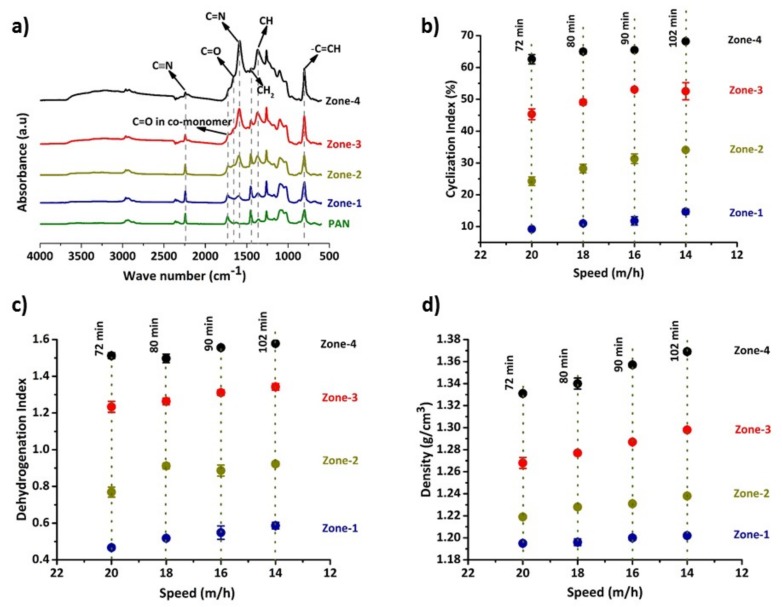
Chemical structure and physical property variations in fibres (**a**) an example FTIR spectra of fibres from each zone of stabilization stage of CF-102 sample, (**b**) variation of cyclization index, (**c**) dehydrogenation index variation, and (**d**) density of samples at each stage with respect to process conditions. Note: Total time taken for stabilization at each speed is represented by vertical dotted lines.

The progress in thermal stabilization of PAN fibres can be assessed by understanding its molecular structural transformations and the variation in the density of fibres. It is known that during thermal stabilization the structural transformations in fibres occur via three chemical reactions 1. Cyclization, 2. Dehydrogenation, and 3. oxidation [28]. Cyclization occurs with the conversion of C≡N to C=N, facilitated by cascade nitrile cyclisation, generating heterocyclic structures. Dehydrogenation is associated with the formation of C=CH from CH–CH_2_ via the loss of hydrogen from the polymer structure, and oxidation results in the formation of carbonyl groups (C=O) and the promotion of dehydrogenation in the polymer backbone [29,30]. The progress of these reactions leads to the evolution of a ladder-like polymer structure and assists in improving fibre thermal stability. Figure 2a shows an overview of the variation of functional groups in fibre structure with respect to thermal treatment at each zone of stabilization stage. In Figure 2a wavenumbers 2243 cm^−1^, 1584 cm^−1^, 1452 cm^−1^ and 1367 cm^−1^ represent the presence of C≡N, combinations of C=N, C=C and NH, CH_2_ and CH functional groups, respectively [31,32]. The wavenumber 1734 cm^−1^ corresponds to the C=O functional group in co-monomers of PAN precursor (typically acrylate derived) [30]. The absorbance at 1661 cm^−1^ represents C=O in conjugated ketones associated with the oxidation reaction [31,33]. 

The peak at 804 cm^−1^ is associated with the C=CH functional group and reflects the occurrence of the dehydrogenation reaction [31]. However, it is difficult to validate the progress of the reaction using this peak due to a coincidence absorbance arising due to the sizing used during precursor manufacture. The progress of oxidation can only be understood qualitatively with respect to time using the C=O peak at 1660 cm^−1^ as the spectra were obtained from a bundle of fibres and the intensity varied with sample quantity. The cyclization and dehydrogenation reactions however, can be quantified using Equations (1) and (2), as they represent the relative variation of C≡N to C=N and CH_2_ to CH functional groups, and thus were used to determine the cyclization and dehydrogenation index.

Figure 2b,c show an increase in the cyclization and dehydrogenation indices during thermal treatment from Zone-1 to Zone-4 at each speed. Similarly, at each zone, samples processed at higher dwell times showed higher values of the indices indicating the increase in thermal stabilization with respect to time. For example, at the end of Zone-4, samples treated for 102 min (14 m/h line speed) exhibited a cyclization index 6% higher than the Zone-4 sample treated for 72 min at 20 m/h line speed. Overall, it is evident that the higher the dwell time, the higher the cyclization and dehydrogenation indices, indicating a higher progress of thermal stabilization. This is further supported by the trends shown by the density of samples from each zone as shown in Figure 2d. These observations suggest that the chemical structural transformations in the polymer backbone promote the increasing compactness in the structure, resulting in denser fibres, which is in good agreement with previous observations [16,18,34]. 

In the final zone of stabilization (i.e., Zone-4) the density of fibres varied from 1.33 to 1.37 g/cm^3^ when fibres were treated for 72 to 102 min. According to previous studies, stabilized fibres with densities between 1.34 to 1.39 g/cm^3^ are better for processing and typically achieve higher tensile properties [13,35,36]. In the current study, stabilized fibres have densities in the suggested range except those fibres treated for 72 min at a line speed 20 m/h, which possessed slightly lower density values (1.33 g/cm^3^). It is interesting to further focus on how the variation in structural transformations of stabilized fibres influences the evolution of planar structures and final properties of carbon fibres, which is detailed in the next section.

### 3.2. Microstructure Evolution in Carbon Fibres

Industrial carbon fibre manufacturing is a continuous process, so any modifications to the line speed not only affects the residence time of fibres in stabilization ovens but also the dwell time in carbonization furnaces. However, the time differences between the respective zones and line speeds is more significant during stabilization compared to the carbonization stage (see Table 2). Hence, in the current study, the basic discussion is focussed on the variation of structure and properties of carbon fibres with respect to dwell times associated with the stabilization stage.

**Figure 3:**
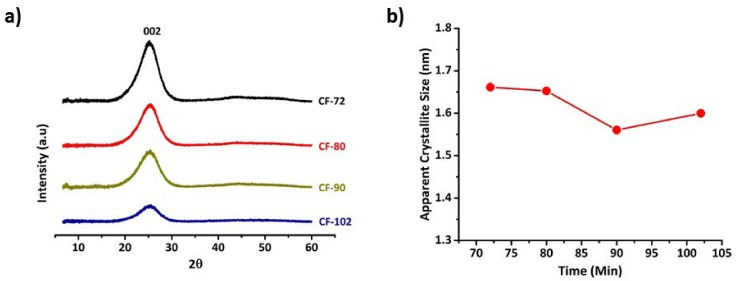
X-ray analysis of carbon fibre samples (**a**) XRD patterns for each samples (**b**) variation of apparent crystallite size between samples.

X-ray studies were conducted on carbon fibres obtained from various residence times during thermal stabilization. Figure 3a shows the XRD patterns of fibre samples, where it is evident that in all the samples there is a peak at diffraction angle ~25.5° corresponding to a crystallographic plane (002) [21,31]. The existence of this peak indicates the evolution of a turbostratic graphitic structure in all carbon fibres. During carbonization, the ladder polymer structure in stabilized fibres is transformed to a planar structures by means of crosslinking with the loss of heteroatoms, usually nitrogen and oxygen [31]. These planar structures stack into layers, due to π–π interactions, and further evolve as crystallites. Based on these peak parameters, an apparent crystallite size (L_c_) of all fibres was calculated using Scherrer’s equation and is shown in Figure 3b. An overall decrease in the apparent crystallite size from 1.661 nm to 1.599 nm for the samples treated for 72 min (line speed: 20 m/h) and 102 min (line speed: 14 m/h) during thermal stabilization was observed. Interestingly, by comparing Figure 2 and Figure 3b, it is evident that the fibres with lower density, cyclization index and stabilized for shorter dwell times possessed higher apparent crystallite size. Also, it is interesting to note that the carbon fibres manufactured at a line speed of 20 m/h spent 3.84 min in carbonization which was less than 14 m/h sample but possessed larger crystallite size. The thermal treatment of PAN fibres at higher dwell times led to a higher cyclization and formation of these cyclic structures have a tendency to promote oxidation in the polymer structure [37]. Moreover, in our previous work the formation of bridging oxygen in the fibre structure was proposed [7], considering the affinity of oxygen towards cyclic structures and our previous proposal, it can be suggested that the fibres treated for 102 min could have higher extent of bridging oxygen atoms which might have hindered the crosslinking mechanism and further led to smaller crystallites during carbonization. Moreover, the structure attained by the fibres treated at 72 min during thermal stabilization should be sufficient to provide more reaction sites for further crosslinking of ladder polymer structures in to planar structures compared to 102 min samples.

### 3.3. Properties of Carbon Fibres

**Figure 4:**
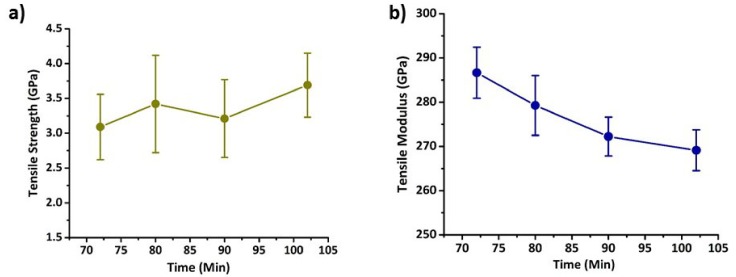
Mechanical properties of carbon fibres with respect to stabilization residence times (**a**) Tensile strength (**b**) Tensile modulus.

The tensile properties of carbon fibres obtained from stabilized fibres treated at various dwell times are shown in Figure 4. From Figure 4a it is appears that the overall tensile strength of carbon fibres increased with increase in residence time, however care needs to be taken in interpreting the results due to the relatively high standard deviation within the sample set. Given the explanation in the Section 3.2 about the ease of formation of planar structures in fibres stabilized at 72 min dwell time compared to fibres thermally treated at 102 min, it can be expected that fibres treated for 72 min should possess higher tensile strength due to the formation of higher amount of crosslinked structures. However, tensile strength of carbon fibres obtained from the fibres thermally stabilized for 102 min showed an average tensile strength 20% higher than the fibres attained from 72 min stabilized fibres. Comparing Figure 3b and Figure 4a it can be observed that higher crystallite size led to a lower tensile strength in carbon fibres. If the crystallite size in carbon fibres is larger it is easy for the crack to propagate and rapidly reach its critical flaw size leading to early fracture and low strength, which could be one of the reasons for lower tensile strength at higher crystallite sizes [38]. Moreover, in the fibres treated above 1250 °C, defects like shearing of crystallites also play a critical role in deciding the tensile strength rather than the surface flaws [38]. In the current scenario, our fibres were also treated at temperatures above 1250 °C. Hence, this could be another reason for showing lower strength in fibres at higher crystallite sizes. 

In contrast, an opposite trend can be observed for the tensile modulus as shown in Figure 4b. The modulus of fibres treated for 72 min during thermal stabilization was 17 GPa higher than the carbon fibres manufactured from precursor stabilized for 102 min. The increase in modulus is generally associated with the orientation of the crystallites in the fibres, however, Liu et al. [38] mentioned that together with the orientation, the apparent crystallite size also play a critical role in influencing the tensile modulus of fibres. Based on the current results, it can be depicted that the increase in modulus is associated with an increase in crystallite size in respective fibres, which is evident by comparing Figure 3b and Figure 4b. Moreover, 72 min of thermal stabilization of PAN fibres for the considered temperature profile could have been enough for the efficient crosslinking of the ladder polymer structures and further might have led to stiffer structures without significantly affecting their orientation during carbonization.

Overall, carbon fibres produced from stabilized fibres whose density is just less than the ideal density range possessed higher modulus and lower tensile strength than the other carbon fibres. From these observations it is evident that there is a trade-off between tensile strength and tensile modulus, which depends on oxidation residence time. Therefore, depending on the desired specifications of the resultant fibre, it is possible to customise a carbon fibre based on dwell time alone. Another parameter which may assist in the evolution of properties during stabilization is the application of tension, which can potentially be used to control the molecular mobility during crosslinking and thus the alignment and growth of the crystallite microstructure. This investigation is currently underway and will be reported in due course.

## 4. Conclusions

The influence of dwell time on the evolution of the structure and property of stabilized fibres and subsequent carbon fibres is presented. During thermal stabilization, fibres which were exposed to stabilisation temperatures at longer times showed higher degrees of polymer cyclization and dehydrogenation, as expected, and attained higher density. These structural transformations resulting from longer thermal stabilization, did not correlate to larger crystallite size during carbonization which was attributed to a lack of crosslinking sites for the formation of graphitic planar structures. However, these smaller crystallite sizes in carbon fibres, attained from longer PAN stabilization, resulted in higher tensile strength. On the other hand, an increase in residence time during thermal treatment showed a decrease in tensile modulus of carbon fibres with the loss of crystallite size. Even though the density of the stabilized fibres was largely maintained in the recommended ideal range (1.34 to 1.39 g/cm^3^), carbon fibres from stabilized fibres with a density of 1.33 g/cm^3^ showed higher tensile modulus and lower strength. Conversely, carbon fibres from stabilized fibres having density 1.37 g/cm^3^ showed a lower tensile modulus and higher strength. Since the carbon fibre manufacturing process involves various parameters, it is difficult to make a generalised statement regarding the requirements of the density of stabilized fibres for attaining high performance carbon fibres. However, density can be a good estimate of the process-ability of stabilized fibres in high temperature carbonization. Based on the findings in this study, it is possible to tailor the resulting carbon fibre properties by merely controlling the dwell time in thermal stabilization stage. This approach also could lead to the production of carbon fibres with higher throughputs and pave the way for the production of low-cost carbon fibres with enhanced mechanical performance. 

## Figures and Tables

**Table 1:** Properties of PAN precursor fibres.

Material	Tensile Strength (GPa)	Tensile Modulus (GPa)	Elongation at Break (%)	Linear Density (dtex)	Diameter (μm)
PAN	0.95 ± 0.06	16.95 ± 0.63	11.69 ± 0.61	0.76 ± 0.09	9.06 ± 0.54

**Table 2:** Process specifications for sample preparation.

Parameters		Stabilization				LT			HT	
		Zone-1	Zone-2	Zone-3	Zone-4	Zone-1	Zone-2	Zone-3	Zone-1	Zone-2
**Temperature (°C)**		228	236	248	258	~500	~800	~1000	~1100	~1500
**Tension (cN)**		~2000	~2100	~2000	~2100	~1100			~1700	
**Line Speed (m/h)**		**Dwell Time (min)**								
**CF-72**	**20**	18	18	18	18	5.4			3.6	
**CF-80**	**18**	20	20	20	20	6			4	
**CF-90**	**16**	22.5	22.5	22.5	22.5	6.75			4.5	
**CF-102**	**14**	25.7	25.7	25.7	25.7	7.7			5.14	

Note: LT: Low temperature carbonization stage; HT: High temperature carbonization stage.
